# Surgical management of aortic regurgitation secondary to Behcet's disease

**DOI:** 10.3389/fcvm.2023.1093024

**Published:** 2023-03-17

**Authors:** Hai-Ou Hu, Chen-Han Zhang, Cristiano Spadaccio, Bing Tang, Cheng-Nan Li, Zhi-Yu Qiao, Tie Zheng, Jun-Ming Zhu, Li-Zhong Sun

**Affiliations:** ^1^ Department of Cardiovascular Surgery, Beijing Anzhen Hospital, Capital Medical University, Beijing Aortic Disease Center, Beijing Institute of Heart Lung and Blood Vessel Diseases, Beijing, China; ^2^ Department of Cardiovascular Surgery, Mayo Clinic, Rochester, MN, United States; ^3^ Department of Cardiovascular Surgery, DeltaHealth Hospital·Shanghai, Shanghai, China

**Keywords:** Behcet's disease, aortic regurgitation, perivalvular leakage, modified Bentall procedure, immunosuppressive therapy

## Abstract

**Background:**

Aortic regurgitation (AR) related to Behcet's disease (BD) is rare, but usually fatal. Perivalvular leakage (PVL) is high if AR related to BD treated with regular AVR. In this study, we report the surgical management of AR secondary to BD.

**Methods:**

Between September 2017 and April 2022, 38 patients with AR secondary to Behcet's disease had surgery in our center. 17 patients did not have a BD diagnosis before surgery, 2 of them were diagnosed during surgery and received Bentall procedure. The remaining 15 patients received conventional AVR. 21 patients were diagnosed as BD before surgery, all of them received modified Bentall procedures. All patients were followed up by regular outpatient visits, transthoracic echocardiogram and CT angiography were performed to evaluate the aorta and aortic valve.

**Results:**

Seventeen patients did not have a BD diagnosis at the time of surgery. Out of them, 15 patients received conventional AVR, and a total of 13 patients suffered PVL after surgery. Twenty-one patients had a BD diagnosis before surgery. They received modified Bentall procedures and IST and steroids were given both pre- and post-surgery. In this group treated with Bentall procedure no patient suffered PVL during follow up.

**Conclusions:**

PVL is a complex scenario after conventional AVR for AR in BD. Modified Bentall procedure seems superior to isolated AVR in these cases. The use of IST and steroids before and after surgery in combination with modified Bentall procedure could have a role in effectively reducing PVL.

## Introduction

Behcet's disease (BD), which was first described by Hulusi Behcet's in 1937, is a chronic systemic inflammatory disorder with imprecise etiology. BD is mainly characterized by recurrent orogenital ulcers and other organ involvement such as skin and eyes (1). According to the current evidence, BD is believed can be triggered by various environmental factors, such as bacterial or viral infections in genetically predisposed individuals (2, 3). Cardiovascular complications is relatively rare in BD, and only 3 to 6% of patients with BD have cardiac complications (4). However, cardiovascular complications related to AR and sinus of Valsalva aneurysms are the leading causes of death in patients with BD (5). In patients suffering from BD, the annulus of the aorta and the aortic walls are fragile due to inflammation of the endothelial tissue (6). The incidence of PVL in BD patients after aortic valve surgery were shown to be higher than those of other diseases, reaching peaks of 40.0%–78.9% (7–9). Different surgical techniques have been suggested to treat BD-related AR in order to reduce the incidence of PVL (6, 10–13), however, there is no consensus on which surgical approach is more favorable. Here we report our experience of surgical management of AR secondary to BD.

## Materials and methods

### Patient enrollment

Between September 2017 and April 2022, 38 patients (29 male) with AR caused by BD underwent surgical repair in our center. Their mean age was 38.3 ± 9.1 years at the time of surgery. Patient characteristics are listed in Table 1. This study was approved by Beijing Anzhen Hospital Institutional Review Board.

**Table 1:** Characteristics of 38 patients with AR related to BD.

First procedure	Value	Pre-IST and steroids	Post-IST and steroids	PVL
AVR	15	0	3	13
Modified Bentall	12	11	12	0
Modified Bentall + MVR	6	5	6	0
Modified Bentall + Arch replacement	4	4	4	0
Modified Bentall + CABG	1	1	1	0

IST, immunosuppressive therapy; PVL, perivalvular leakage; AVR, aortic valve repair; MVR, mitral valve replacement; CABG, coronary artery bypass grafting.

Seventeen patients did not have a BD diagnosis at the time of surgery, 2 of them were diagnosed during surgery (1 patient received Bentall procedure, the other received Bentall + MVR) and IST and steroids were given after surgery. The remaining fifteen patients received conventional AVR, 3 of them were suspected with BD during operation, IST with steroids were given after surgery, even though they were not diagnosed as BD according to ISG criterion (14). The other 12 patients not diagnosed or suspected with BD received conventional AVR, IST and steroids were not given after surgery. All these originally undiagnosed patients eventually received a definite BD diagnosis.

Twenty-one patients were diagnosed as BD in accordance with ISG criterion (14) before surgery, they received modified Bentall procedures, and all the patients were received IST and steroids before and after surgery.

Concomitant conditions included 6 patients with mitral insufficiency (MI), 7 patients with aortic root aneurysm, 4 patients with aortic arch aneurysm, aortic valve perforation was found in 4 patients, interventricular septal dissection was found in one patient, sub-annular cyst was found in 5 patients. One patient was suspected with aortic valve vegetation, but no bacteria were found in the culture.

### Surgical techniques

In our institution, patients with unsuspected or undiagnosed BD receive conventional AVR for the treatment of AR. Patients with defined diagnosis of BD as per ISG criteria are treated with modified Bentall.

The procedure was performed under general anesthesia through a median sternotomy. In re-operation cases, the femoral artery and vein were prepared before the median sternotomy in case of the need of prompt CPB initiation. Histidine-Tryptophan-Ketoglutarate (HTK) solution was antegrade administered into the coronary ostia. The coronary ostia were detached as a button with a 1 cm diameter cuff of the aortic wall, and mobilized over a short length to facilitate reimplantation. The aortic root was dissected to the level of annulus, an 8 mm width Teflon felt was placed and it immediately encircled the aortic root. Interrupted mattress sutures were used, passing from outside to inside through the felt strip, the aortic wall and the sewing ring of the prosthetic valved graft (SJMTM Masters Series Aortic Valved Graft, Abbott Medical Canada, Inc.) sequentially. By so doing, the aortic wall was sandwiched between the external Teflon felt and the sewing ring of the prosthetic valve. Then the sutures were tightened to secure the prosthetic valve at the level of annulus. Then the coronary ostia were anastomosed to the valved graft in an end-to-side fashion with continuous running suture with 5-0 propylene. At last, the distal anastomosis was done with running suture in an end-to-end fashion. Concomitant procedures were carried out, including MVR in 6 patients, aortic arch replacement in 4 patients, and CABG in 1 patient.

### Medication and follow-up

The median follow-up time was 34.5 months (4.1–98.1 months).

For patients diagnosed with BD before surgery, vasculitis was controlled with IST (such as cyclophosphamide) and steroids, the level of ESR and CRP were found to be in normal range, MRA showed no active vasculitis. For patients who were diagnosed with BD before or during surgery, or patients who was suspected with BD during surgery, IST and steroids were given immediately after admission to intensive care unit. Hydrocortisone was given intravenously for 3 consecutive days. After that, oral administration of prednisolone (10 mg per day) was begun.

After discharge, the patients were followed up by regular outpatient visits. Transthoracic echocardiogram and CT angiography were performed to evaluate the aorta and aortic valve. Patients were also recommended to consult a rheumatologist regularly to control inflammation and change the immunosuppressive regimen and steroids when necessary.

## Results

Out of the seventeen patients without BD diagnosis at the time of surgery, a total of 13 patients suffered PVL. The median time interval between first AVR and PVL was 5 months (4 weeks to 18 months). The mean aortic root diameter was 37.38 ± 5.71 mm in patients diagnosed with BD before the surgery, and 35.29 ± 6.68 mm in patients who were not diagnosed with BD. More details of echocardiographic data and inflammation parameters were shown in Tables 2, 3. Twelve patients received conventional AVR, with no IST and steroids after surgery and all suffered PVL. Three patients were suspected as BD during the operation and IST was given after surgery. Out of these 3 patients only one patient suffered PVL. Two patents were diagnosed with BD during surgery, modified Bentall procedures were carried out, IST and steroids were given after surgery according to rheumatologist's suggestion and they did not have PVL during follow up.

**Table 2:** Echocardiographic data.

	Before surgery			After surgery		
Variants	Diagnosed group	Undiagnosed group	*P* value	Diagnosed group	Undiagnosed group	*P* value
Aortic root diameter (mm)	37.38 ± 5.71	35.29 ± 6.68	0.31	27.15 ± 4.31	28.07 ± 4.5	0.55
Ascending aorta diameter (mm)	36.76 ± 6.57	36.47 ± 4.62	0.88	26.38 ± 4.32	27.47 ± 5.18	0.50
Pulmonary artery diameter (mm)	25.71 ± 4.10	24.00 ± 3.45	0.18	23.61 ± 3.73	23.06 ± 3.21	0.65
Left atrial diameter (mm)	50.43 ± 9.79	49.47 ± 8.25	0.75	36.6 ± 9.95	44.25 ± 13.52	0.06
IVST (mm)	10.21 ± 1.74	10.31 ± 2.03	0.86	10.98 ± 2.46	10.47 ± 1.89	0.52
IVSE (mm)	8.80 ± 2.12	8.19 ± 1.76	0.36	5.29 ± 2.82	4 ± 3.3	0.24
LVEDD (mm)	64.90 ± 5.69	59.82 ± 8.56	0.04	50.81 ± 8.81	47.44 ± 10.03	0.28
Right ventricular diameter (mm)	21.76 ± 3.90	20.56 ± 5.34	0.46	19.89 ± 2.22	19.47 ± 3.62	0.68
RVOT (mm)	26.39 ± 3.60	24.53 ± 3.78	0.16	25.33 ± 4.03	23.44 ± 3.16	0.14
Ejection fraction (%)	60.24 ± 6.48	61.06 ± 5.55	0.68	52.05 ± 11.11	54.94 ± 11.69	0.45
LVFS (%)	32.24 ± 4.68	34.00 ± 3.40	0.22	28.6 ± 5.01	31.75 ± 4.79	0.11
Peak flow velocity (m/s)	224.86 ± 59.50	251.94 ± 98.06	0.30	231.95 ± 59.18	209.38 ± 34.9	0.18
AVPG (mmHg)	5.20 ± 1.56	5.55 ± 3.46	0.41	22.45 ± 11.09	18.87 ± 4.96	0.25
SPAP (mmHg)	40.17 ± 13.44	33.60 ± 11.28	0.23	28.44 ± 7.42	31.7 ± 14.13	0.54
Regurgitant area (cm^2^)	15.15 ± 4.80	11.62 ± 2.97	0.45			

IVST, interventricular septal thickness; IVSE, interventricular septal excursion; LVEDD, left ventricular end-diastolic dimension; RVOT, right ventricular outflow26. tract; LVFS, left ventricular fractional shortening; AVPG, aortic valve pressure gradient; SPAP, systolic pulmonary artery pressure.

**Table 3:** Baseline data.

Variants	Diagnosed group	Undiagnosed group	*P* value
C-reactive protein (mg/L)	5.59 ± 8.12	7.42 ± 12.28	0.66
Erythrocyte sedimentation rate	12.53 ± 15.81	10.82 ± 8.55	0.70
Complement 3 (g/L)	1.04 ± 0.19	1.11 ± 0.11	0.34
Complement 4 (g/L)	0.24 ± 0.09	0.24 ± 0.1	0.96
Immunoglobulin A (g/L)	2.09 ± 0.83	2.37 ± 0.71	0.52
Immunoglobulin G (g/L)	9.55 ± 3.7	10.63 ± 3.12	0.58
Immunoglobulin M (g/L)	0.78 ± 0.38	1.02 ± 0.56	0.35
White blood cell count ( × 10^9^/L)	7.03 ± 2.35	7.24 ± 2.55	0.80
Red blood cell count ( × 10^9^/L)	4.28 ± 0.64	4.2 ± 0.54	0.71
Haemoglobin (g/L)	131.21 ± 21.23	129.82 ± 16.26	0.83
Platelet count ( × 10^9^/L)	209.16 ± 58.07	205.12 ± 49.94	0.83
Prothrombin time (s)	14.87 ± 6.25	15.11 ± 6.6	0.91
International normalized ratio	1.31 ± 0.52	1.31 ± 0.55	1.00
D-Dimer (ng/ml)	90 ± 50.35	146.41 ± 175.91	0.20
Aspartate transaminase level (U/L)	36.65 ± 79.31	19.35 ± 12.36	0.38
Alanine transaminase level (U/L)	28.25 ± 42.52	18 ± 6.42	0.33
Total Protein (g/L)	64.38 ± 6.78	65.72 ± 4.68	0.50
Albumin (g/L)	38.7 ± 9.43	39.02 ± 9.89	0.92
Total bilirubin (*μ*mol/L)	16.5 ± 9.09	13 ± 9.01	0.27
Direct bilirubin (μmol/L)	4.16 ± 1.89	4.38 ± 3.12	0.80
Blood urea nitrogen (mg/dl)	6.3 ± 1.94	6.28 ± 2.2	0.98
Serum creatinine (μmol /L)	74.68 ± 13.96	71.28 ± 13.82	0.46
Uric acid (μmol /L)	419.7 ± 96.67	374.75 ± 84.32	0.14
Glucose (mmol/L)	4.75 ± 0.74	4.65 ± 0.6	0.68
Sodium (mmol/L)	141.93 ± 1.88	140.4 ± 2.4	0.04
Potassium (mmol/L)	3.92 ± 0.3	4.12 ± 0.45	0.12
Creatine kinase (μg /L)	49.74 ± 25.64	54.13 ± 27.58	0.63
Lactic dehydrogenase (μg /L)	290.47 ± 352.32	230.63 ± 102.15	0.52
CK-MB (μg /L)	1.78 ± 1.41	1.21 ± 0.61	0.14
Troponin I (μg/L)	0.14 ± 0.23	0.41 ± 0.69	0.56
Myoglobin (μg /L)	31.05 ± 25.39	19.17 ± 2.83	0.44
pH	7.42 ± 0.03	7.42 ± 0.03	0.91
Partial pressure of oxygen (mmHg)	100.03 ± 25.41	97.19 ± 12.35	0.69
Partial Pressure of Carbon Dioxide (mmHg)	36.45 ± 3.26	36.58 ± 4.19	0.92
Lactic acid (mmol/L)	1.96 ± 0.71	1.75 ± 0.53	0.35

Redo operations were carried out for these patients who suffered PVL (Table 4). Two patients received redo AVR, one patient suffered PVL again and received a third operation (modified Bentall procedure) with no further PVL during follow up. One patient received percutaneous interventional closure because of high surgical risk. Ten patients received modified Bentall procedures as described and no PVL were found during follow-up. Two patients suffered complete atrioventricular block (CAVB) after reoperation, one was implanted with permanent pacemaker, the other progressed to II degree and no permanent pacemaker was implanted. One patient died because of low cardiac output on post operation day 3, the other patients were all alive at follow-up.

**Table 4:** Clinical data of patients with PVL secondary to AVR.

No.	Sex	Age	First operation	PVL time (months)	Complications	Re-operation	Complications
1	M	43	AVR + Sinus repair	6	PVL	Modified Bentall + LVOT Rec	–
2	F	37	AVR	2	PVL	Modified Bentall	–
3	M	42	AVR + MVR	3	PVL	AVR	PVL
4	M	18	AVR	4	PVL	Modified Bentall	AVB(III→II)
5	M	31	AVR	1.5	PVL	Modified Bentall	–
6	M	33	AVR	6	PVL	Modified Bentall	–
7	F	28	AVR	18	PVL	Modified Bentall	–
8	M	41	AVR	11	PVL	Modified Bentall	AVB (III)
9	M	43	AVR	1	PVL	Interventional closure	PVL
10	M	38	AVR	6	PVL	Modified Bentall + LVOT Rec	–
11	M	47	AVR	14	PVL	Modified Bentall	–
12	M	40	AVR	5	PVL	Modified Bentall	–
13	M	31	AVR	3	PVL	AVR + MVR + LVOT Rec	–

AVB, atrioventricular block; AVR, aortic valve replacement; LVOT, left ventricular outflow tract; MVR, mitral valve replacement; PVL, perivalvular leakage; Rec, reconstruction.

Twenty-one patients diagnosed with BD before surgery received IST and steroids before and after surgery, none of them suffered PVL during follow up.

## Discussion

Behcet's disease (BD) is an inflammatory disease with unknown etiology, and characterized by recurrent flares which could affect the mucocutaneous tissues, eyes, joints, blood vessels, brain, and gastrointestinal tract.

The natural history of BD includes periods of exacerbations and remissions, though the pathogenesis is still unclear, many studies had shown a same path: an abnormal immunopathological process with plenty of triggers, such as inflammation, genetic predisposition linked to the HLA-B51 allele (15). Typical characteristics of BD include vascular injuries, hyperfunction of neutrophils, and autoimmune responses are (1). Vasculitis in BD is neutrophil-predominant, affecting all layers of the vessel and vasa vasorum, which can present fibrous thickening and nonspecific inflammatory infiltrate in the late phase (16).

Neutrophils could increase superoxide production, enhanced chemotaxis, and excessive production of lysosomal enzymes in patients with BD, which indicates neutrophils are overactive, and eventually lead to tissue damages (17, 18).

Cardiac complications are relatively rare in BD patients, which mainly include pericarditis, myocarditis, endocarditis with valvular regurgitation, intra cardiac thrombosis, endomyocardial fibrosis, coronary arteries aneurysms, and sinus of Valsalva aneurysm (19). In BD patients who have a destructed aortic valve, both the aortic annulus and the ascending aorta are affected by the disease. The persistent inflammatory reactions induce fragility in both the aortic annulus and the ascending aortic wall, which finally lead to PVL after aortic valve replacement. The inflammatory process in the aortic wall can also causes destruction of elastic fibers, leading to transmural necrosis of the aortic walls in major arteries, with consequent formation of aneurysms (16).

Isolated aortic valve replacements (AVR) were performed to those patients who had severe aortic regurgitation (AR) associated with BD in the past (20). However, isolated AVR was reported with high mortality and re-operation rates (20% to 47.3% and 78% to 100%, respectively) (7, 8, 21).

Jeong et al. reported the outcomes of 19 BD patients during a 22-year follow-up period. Four patients underwent aortic root replacement, 14 patients underwent AVR and 1 patient underwent AVP. Overall mortality was 47.3%. Fifteen patients (78.9%) required re-operation because of valve detachments (7). Due to the fragility of aortic tissue and persistent inflammation, BD patients underwent isolated AVR have poor outcomes, paravalvular leakage, valve detachment, and pseudo-aneurysms were seen in many patients after the surgery (8).

In this study, 13 out of 17 patients with undiagnosed BD underwent isolated AVR and suffered PVL after surgery with a median time of 5 months (1 month to 18 months), including 1 patient who was suspected as BD during operation with IST and steroids given after surgery.

To reduce theses complications, different surgical techniques were required, such as modified AVR, modified Bentall procedure, etc.

Takashi et al. performed a novel modified AVR called “sub-annular ring reinforcement technique” in three patients, with no prosthetic valve detachment after a mean period of 3 ± 1.8 years (22). Compared with isolated AVR, aortic root replacements may lead to better outcomes (6, 23). Japanese researchers have reported that the incidence of valve detachment is much lower in patients who underwent a translocated Bentall procedure or valved conduit procedures (a modified Bentall operation) than those who underwent isolated AVR (8, 24). The modified Bentall procedure which uses a sub-annular inverted graft and rapid deployment valve would be useful to prevent prosthesis dehiscence in patients with BD (10).

In our study, once BD was diagnosed before surgery, modified Bentall procedure would be performed as a standard. Using this modified Bentall procedure, no PVL has been found in either the first operation or reoperation. The possible reason is all anastomosis were strengthened by performing modified Bentall procedure.

CAVB is a common complication of BD-related AR, the incidence was 48.6%, whereas the incidence of CAVB which requiring pacemaker implantation after the surgery in patients with general AR was reported to be 3.2% (25). The potential cause of high incidence of CAVB is the location of atrioventricular node, which is just below the non-coronary cusp of atrioventricular, the persistent inflammatory reactions may also affect the atrioventricular node (26).

In this study, none of the patient suffered CAVB at the first operation. For the 13 re-operation patients, 2 patients suffered CAVB, one of them was implanted with permanent pacemaker, the other improved to II-degree block, and no pacemaker was implanted.

Beside surgical strategies, immunosuppressive and steroids therapy also played a very important role in BD patients.

It has been reported that IST may improve the peri-operative outcomes, and should be started before the surgery. In patients who received the steroids therapy before the surgery, inflammatory reactions were obviously less severe, in these patients, lymphocytic infiltration in the aortic wall and aortic valve was the predominant finding. However, in patients not administered preoperative steroid therapy, the major infiltrated cells were neutrophils, suggesting the acute inflammatory reaction (7).

Immunosuppression and/or immunomodulation therapies were also deemed as essential treatments to the BD patients (2). Guo reported that the PVL is much more common in BD patients who underwent isolated conventional AVR (47% at a median interval of 3.5 months), and preoperative immunosuppressive therapy, especially cyclophosphamide in combine with glucocorticoid, could reduce the incidence of PVL after the surgery and improve the outcomes (27).

Li reported that IST can significantly improve the prognosis of BD patients with aortic valve regurgitation and/or arteritis. However, there was no significant difference in the incidence of re-operation or death between patients with peri-operative IST and postoperative IST alone (28). Choi also reported that postoperative IST was a protective factor in the development of PVL (29).

In this study, 21 patients were diagnosed as BD before the surgery, IST and steroids therapy were started according to rheumatologist's suggestion. These patients received modified Bentall procedure and postoperative IST and steroids therapies, none of them suffered PVL after the surgery. In three patients suspected with BD during the operation, isolated conventional AVR was performed, IST and steroids therapy were started immediately after the surgery. Only one of them developed PVL. In two patents diagnosed with BD during the operation, modified Bentall procedure was performed, IST and steroids therapy were given after the surgery, none of them developed PVL during the follow up. Modified Bentall procedure could provide a more favorable outcomes for BD patients. At the same time, pre-operative IST and steroids therapy are also very important for BD patients in preventing PVL.

Finally, it is important to keep in mind that severe AR can be an initial manifestation of BD even in patients who do not meet the current diagnostic criteria. Surgeons should be cognizant of the possibility of cardiovascular BD in young patients with AR of unknown etiology before performing first-time aortic valve replacement, or in cases of of PVL, prosthetic valve detachment and pseudo-aneurysm after aortic valve replacement.

Authors acknowledge several limitations in this study. Firstly, this was a retrospective cohort study and it shares all the known limitations of observational reports.

Secondly, in our cohort, all the patients who were diagnosed with BD before surgery received IST and steroids, and modified Bentall procedure, while patients initially undiagnosed were treated according to standard protocols with isolated AVR and no further IST and steroids. Clearly this precluded any form of meaningful comparison between the patients initially undiagnosed and the ones who had a definite diagnosis at the time of surgery. However, this report was not designed as a comparative study or powered to identify predictive factors. The conclusions drawn are only based on a cohort observation and cannot be considered definitive, but only hypothesis-generating. Ad hoc comparative studies should be performed to elucidate these points.

## Conclusion

Early diagnosis of BD-related AR, especially in patients not satisfying the current diagnostic criteria is crucial and surgeons should be cognizant of the possibility of BD in young patients with isolated AR or recurrent PVL or valve detachment after AVR. In our experience, younger ages (younger than 50 years old), history of multiple ulcers, AR without specific reasons such as degenerative disease or rheumatic heart disease, and rapidly progressive PVL after AVR are highly associated with BD, these patients are recommended to undergo further examination. According to our data, the combined use of modified Bentall procedure and IST before and after surgery in BD patients might effectively reduce the occurrence of PVL and should be recommended.

## Data Availability

The raw data supporting the conclusions of this article will be made available by the authors, without undue reservation.
